# Novel Response Surface Technique for Composite Structure Localization Using Variable Acoustic Emission Velocity

**DOI:** 10.3390/s24113450

**Published:** 2024-05-27

**Authors:** Binayak Bhandari, Phyo Thu Maung, Gangadhara B. Prusty

**Affiliations:** 1 School of Mechanical and Manufacturing Engineering, University of New South Wales, Sydney, NSW 2052, Australia; ptmaung90@gmail.com (P.T.M.); g.prusty@unsw.edu.au (G.B.P.); 2 Australian Composite Manufacturing CRC Ltd., Sydney, NSW 2052, Australia

**Keywords:** acoustic emission, composite laminates, localization, least-square method, response surface, velocity attenuation, time difference of arrival

## Abstract

The time difference of arrival (TDOA) method has traditionally proven effective for locating acoustic emission (AE) sources and detecting structural defects. Nevertheless, its applicability is constrained when applied to anisotropic materials, particularly in the context of fiber-reinforced composite structures. In response, this paper introduces a novel COmposite LOcalization using Response Surface (COLORS) algorithm based on a two-step approach for precise AE source localization suitable for laminated composite structures. Leveraging a response surface developed from critical parameters, including AE velocity profiles, attenuation rates, distances, and orientations, the proposed method offers precise AE source predictions. The incorporation of updated velocity data into the algorithm yields superior localization accuracy compared to the conventional TDOA approach relying on the theoretical AE propagation velocity. The mean absolute error (MAE) for COLORS and TDOA were found to be 6.97 mm and 8.69 mm, respectively. Similarly, the root mean square error (RMSE) for COLORS and TODA methods were found to be 9.24 mm and 12.06 mm, respectively, indicating better performance of the COLORS algorithm in the context of source location accuracy. The finding underscores the significance of AE signal attenuation in minimizing AE wave velocity discrepancies and enhancing AE localization precision. The outcome of this investigation represents a substantial advancement in AE localization within laminated composite structures, holding potential implications for improved damage detection and structural health monitoring of composite structures.

## 1. Introduction

Advances in information and sensor technologies have made structural health monitoring (SHM) possible using a data-driven approach. Nondestructive testing (NDT) is preferred over destructive testing (DT) for early deterioration signs because it offers a safe and reliable inspection that is economical and can be performed without disrupting ongoing operations [1]. Further, NDT methods use techniques that do not necessitate permanently destroying the structure or system for evaluating, detecting, and collecting data. The most commonly used NDT techniques include visual inspection, ultrasonic [2], magnetic particle, radiography [3], and acoustic emission [4].

Acoustic emission (AE) is a phenomenon that occurs under various conditions and not only limited to stress or strain conditions; for example, phase transformation, corrosion, acoustic excitation from the environment, etc. In such a case, energy is released from material in the form of elastic waves (or acoustic waves). AE sources are generated because of plastic deformation, crack propagation, fiber breakage, de-bonding, impact, leakage, and friction. One phenomenon leads to another, such as crack initiation, which might be caused by plastic deformation which in turn can be due to aging, thermal gradients, or simply because of mechanical loadings. The AE technology is a mature NDT technique that uses piezo-electric transducers for examining the behavior of the structure under stress. Identification of the AE event location can assist in implementing other NDT techniques in the close vicinity of the source for further investigation.

The main objectives of conducting an AE test on a structure are to identify, assess, and keep track of any defects present in the structure that may pose a risk to its structural integrity. The basis of source location is to attach a network of sensors on the surface of the structure, the AE signal arrival time for each sensor is recorded, and the time difference is determined. Based on the known acoustic velocity of the tested material, it is possible to locate the AE event using simple triangulation methods [5]. This method is known as the time of arrival (TOA) technique [6,7]. However, on many occasions, the exact time of the AE event is difficult to determine upfront; instead, the AE hits are picked-up by sensors and time-recorded. Once the AE event signal is received at sensors (at least three sensors for 2D panels and four sensors for 3D structures), the difference in arrival time can be used to calculate the differences in distances between the AE source and sensors. This method is known as the Time Difference of Arrival (TDOA) or delta T source location [8] method, which is more versatile than TOA.

Madarshahian et al. [9] proposed an approach to select the most probable onset time in an AE signal for locating the AE event source using two Bayesian framework methods. They evaluated their approach in the concrete structure. Zhou et al. [10] proposed the AE source location method considering the refraction index in two media. Their study used sensor coordinates, the arrival time of acoustic waves, and the velocities of acoustic waves for two media.

In the realm of AE-based localization, numerous studies have been carried out to tackle diverse applications [11,12,13]. These investigations encompass crack detection in a fluid pipeline [14], zonal localization on steel plates [15], concrete structures [16], asphalt mixtures [17], outer race of bearings [18], and riveted metallic panels [19]. However, a notable trend across these studies is their exclusive focus on structures with isotropic materials.

Localization of the AE events in anisotropic laminated composite structures is an active research area. Eaton et al. [20] conducted a thorough investigation on the “Delta T mapping” source location methodology proposed by Baxter et al. [8], which was originally developed for use in isotropic materials. Sikdar et al. [21] carried out an AE source localization using experimental and finite element analysis in a honeycomb sandwich structure. Kundu et al. [22] used a two-step technique to determine the AE source in an anisotropic structure. In the first step, the approximate AE source coordinates and two AE velocities were estimated. In the second step, a more accurate estimation of source coordinates and two wave speeds were performed by minimizing the objective function using the simplex method. These studies used lead–zirconium–titanium oxide (PZT) as the AE sensor.

Fiber-optic Bragg gratings (FBGs) were also used as AE sensors for AE source localization and subsequent analysis for damage progression in carbon fiber-reinforced plastic (CFRP) laminates by [23,24]. Yu and Okabe [23] used a single Fiber-optic Bragg grating (FBG) as the AE sensor for damage localization in CFRP laminates. Jang and Kim [25] implemented a triangulation method and neural network to enhance the fiber optic sensor signal for impact localization in composite stiffened panels. 

Unlike conventional machine learning algorithms, deep learning (DL) techniques are referred to as “end-to-end” learning approaches, indicating that they perform feature extraction, classification, and regression in a sequential manner. Therefore, DL methods are considered “black-box” models. Machine learning (ML) and deep learning (DL) algorithms have been widely applied to the AE source location, degradation detection problems like corrosion detection [26,27], and monitoring cracks in large-scale orthotropic steel plates [28]. In this line, Sikdar et al. [29] and Bhandari et al. [30] proposed a CNN-based deep-learning framework for predicting damaged regions in CFRP panels. They converted time-domain AE signals to time-frequency scalograms images by performing continuous wavelet transform (CWT), which was used as input to the CNN model. Liu et al. [31] proposed a generalized regression neural network acoustic source localization method based on time difference mapping (GRNN-TDM). They used time-difference of the sensor path as the training input data for the neural network, while the output was the coordinates of grid nodes. More recently, Mahajan and Banerjee [32] used an AE source localization technique based on deep learning for monitoring rail sections, where they normalized the signal data and used continuous wavelet transform (CWT) to generate high-resolution RGB images.

In the field of AE, extensive research has been conducted to explore various techniques and their advantages/disadvantages. Kundu [33] provided a comprehensive review of these localization methods, including the beamforming technique extended to anisotropic structures [34,35], which requires prior knowledge of the direction-dependent velocity profile. Kundu et al. [36,37] attempted to address this issue, introducing a technique for source localization that circumvents the need for prior velocity profile information. More recently, comprehensive reviews by Ma et al. [38] have encapsulated the breadth of Acoustic source localization (ASL) techniques and innovative approaches like deep learning, time reversal, and energy-based methods-primarily contrasting their effectiveness around the use of L-shaped sensor clusters, where wave path and wavefront geometries are critical factors. 

Despite these advances, the dynamics of the AE signal transmission, including the nuances of signal attenuation [39], wave velocity changes [40], and the influence of material fiber orientation [5] on the AE wave propagation, remain insufficiently studied [41]. The prevailing approaches often rest on two fundamental assumptions: wave speeds are constant, and the trajectory between the sensor and AE source is a straight line. While the latter can be reasonably accepted for structures with uniform thickness, the former presents a significant divergence from what is observed in practical engineering materials, especially laminated composites.

To bridge this gap, we present a two-step AE source localization technique that elucidates the wave velocity attenuation and angular propagation. This approach meticulously constructs a response surface model that accounts for the complexities of the AE wave velocity variations, attenuation rates, sensor-to-source distances, and fiber orientation. In the first step, we developed the response surface from empirical data, capturing the aforementioned parameter interplay. Following this, the refined velocity parameters are utilized within a tailored algorithm that boasts enhanced accuracy in pinpointing the AE sources. Our meticulous method surpasses the limitations of conventional time-of-arrival (TOA) calculations, as evidenced by comparative analysis, which underscores its strength in reliably detecting AE events within composite laminates.

Evidently, the proposed method has substantial implications for the field of nondestructive testing and health monitoring of composite structures, providing a robust tool that overcomes the shortcomings of existing methodologies.

## 2. Materials and Methods

### 2.1. Laminated Composite Panel Manufacturing

Eight layers of unidirectional thermoplastic carbon-fiber/PEEK prepregs (supplied by Solvay) were laid in a quasi-isotropic layup of [0/+45/−45/90]s before being placed under the hot-press machine (IDM instruments) to fabricate a carbon-fiber reinforced polymer (CFRP) laminated panel.

Quasi-isotropic laminates offer several advantages, including uniform stiffness and isotropic behavior in all directions within the plane of the laminate. Additionally, the symmetry and balance in ply orientations simplify the manufacturing process, making fabrication easier. Quasi-isotropic laminates are also commonly used for benchmarking, standardization testing procedures, and material databases, making them an ideal choice for our experiment.

The prepregs were hot-pressed according to the manufacturer’s recommended curing temperature and time. The edges of the cured composite panel were then precisely cut into the required dimensions of 300 mm × 300 mm using the Axitom-5 diamond-saw cutting machine. Afterwards, the panel was marked into a 250 mm × 250 mm test area with 50 mm gridlines and an edge offset of 25 mm. 

**Figure 1:**
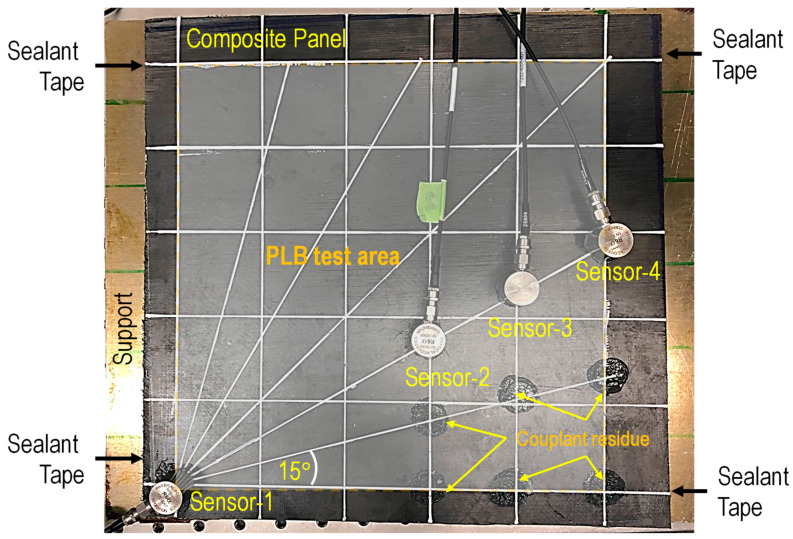
An experimental setup with sensors and pencil lead break test area.

Hsu–Nielsen AE tests or pencil lead break (PLB) tests were carried out at the test area, as shown in Figure 1, to characterize the acoustic wave propagation in a composite panel. The edge offset was applied to minimize the influence of reflected AE signals from the edges. Sealant tapes (not visible) were placed in a number of places between the composite panel and the metallic support to isolate the panel from the metallic support. Also, the figure shows the sensor location on a straight line inclined at 15° intervals from 0° to 90° for determining AE wave velocity. Also seen in the figure is the couplant residue left after the sensor was removed for the third time, following two previous tests. The horizontal, vertical, and inclined lines are reference lines for conducting experiments.

### 2.2. Acoustic Emission Test Hardware and Software

The AE system used in this study includes AE hardware, sensor, preamplifier, and software. The details of each component are as follows: a Micro-II Digital AE system with eight channels from Physical Acoustics Corporation (USA) [42] and lead–zirconium–titanium oxide (PZT) R6α—60 kHz general purpose AE sensor [43] was used in this study.

In order to amplify the very low AE signal amplitude (voltage) generated by the AE sensors into a usable form, MISTRAS voltage preamplifiers with a gain of 40 dB were used for each channel. Finally, AEwinTM Windows^®^-based program [44] was used for real-time AE data acquisition and waveform processing, display, and data storage.

Pencil-lead breakage (PLB) was utilized as a reproducible artificial acoustic emission (AE) source, which is also referred to as the Hsu–Nielsen source [45,46]. For each PLB test, stress waves that travel along the surface of the panel were captured by AE sensors. AE signal data (CSV file) from the MISTRAS system was used as the source of the AE data. Data cleaning, data extracting, and feeding to the optimization program were carried out using an in-house program written in Python. The arrival time of the AE signal was determined by calculating the peak waveform of the detected wave for each sensor.

### 2.3. Sensor Layout and Data Acquisition

The objective of most AE structural investigations is to monitor emissions from all regions where failures could occur. In the most basic form, a single AE sensor can report the behavior of structure on varying loading conditions. However, in an actual engineering problem, the flaw can originate from many possible sites, requiring multiple AE sensors.

In this study, the experiments were conducted in a panel as described in Section 2.1. The PLB tests were well within the acoustic range of all four sensors used in the study.

**Figure 2:**
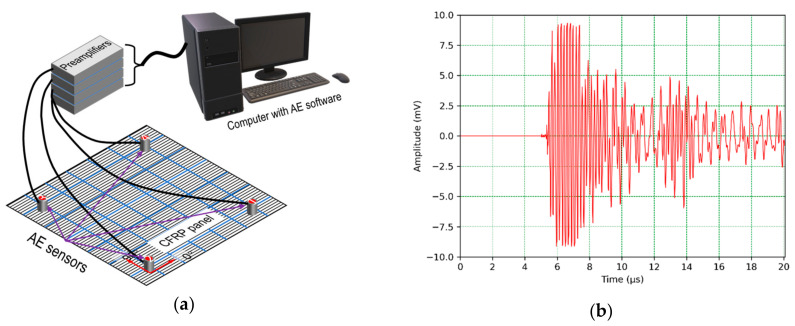
(**a**) Experimental setup with AE sensors and computer system and (**b**) typical AE wave signal received by a sensor.

After the AE equipment was configured and setup complete, as shown in Figure 2a, the AE sensors were held in position to the test surface by an acoustic couplant layer of silicone grease. Almost all of the AE signals were shorter than 20 µs because of the high attenuation of the composite panel. Figure 2b shows the typical AE wave signal in the CFRP composite panel.

## 3. AE Localization Principles

The basic idea of locating an AE source in metallic structures is three-fold; first, attaching a number of AE sensors on the surface; second, determining the AE signal arrival time to each sensor; and finally, triangulating back the location of the AE source based on the arrival time and AE velocity. In the above steps, several simplifications are made:

- constant theoretical acoustic wave velocity is used;
- the shortest path (or a straight line) between the AE source and sensor is considered;
- for triangulation, the set of nonlinear equations can be easily solved.

A concise theoretical background of the AE localization problem [47] using the TDOA technique is presented below. The relation among the velocity of acoustic wave (*v*), the distance between the sensor to the AE source (*d*), and arrival time (*t*) can be written as:

(1) d=v×t

the Euclidean distance between two points *p* and *q* in a x–y plane is given by:

(2) $$d\left(p,q\right)=\sqrt{{\left(x_{2}-x_{1}\right)}^{2}+{\left(y_{2}-y_{1}\right)}^{2}}$$

The exact time the AE event occurred is unknown; therefore, to simplify the problem, all the times are considered relative to the first AE signal to reach the sensor. The time difference between the second hit relative to the first hit $\left(t_{2}-t_{1}\right)$ can be written as:

(3) $$t_{2}-t_{1}=\frac{\left(d_{2}-d_{1}\right)}{v}$$

Equation (2) combined with the Equation (3) yields:

(4) $$t_{2}-t_{1}=\frac{\sqrt{{\left(x_{2}-x_{s}\right)}^{2}+{\left(y_{2}-y_{s}\right)}^{2}}-\sqrt{{\left(x_{1}-x_{s}\right)}^{2}+{\left(y_{1}-y_{s}\right)}^{2}}}{v}$$

where x_s_ and y_s_ are the unknown coordinates of the AE source. Equation (4) contains two unknowns and cannot be solved by the general procedure. A similar approach for an AE sensors network with multiple sensors provides more data, which leads to better AE source prediction. Therefore, the general formula for time difference can be written as

(5) $$t_{i}-t_{1}=\frac{\sqrt{{\left(x_{i}-x_{s}\right)}^{2}+{\left(y_{i}-y_{s}\right)}^{2}}-\sqrt{{\left(x_{1}-x_{s}\right)}^{2}+{\left(y_{1}-y_{s}\right)}^{2}}}{v}$$

Equation (5) essentially calculates the same time difference (Δt) of the ith sensor using two methods. The left hand of Equation (5) calculates the observed time difference, Δ_t,obs_, using the known arrival times, whereas the right-hand side of Equation (5) defines the calculated time difference Δ_t,calc_. A general-purpose multiple regression algorithm is able to minimize the difference between Δ_t,obs_ and Δ_t,calc_ values. The difference between an observed value and a calculated value (in parentheses) in a regression model is called the residual. To understand how well a regression model fits a dataset, the residual sum of squares is calculated as shown in Equation (6).

(6) $$\chi^{2}=\sum {\left(\Delta t_{i,obs}-\Delta t_{i,calc}\right)}^{2}$$

**Figure 3:**
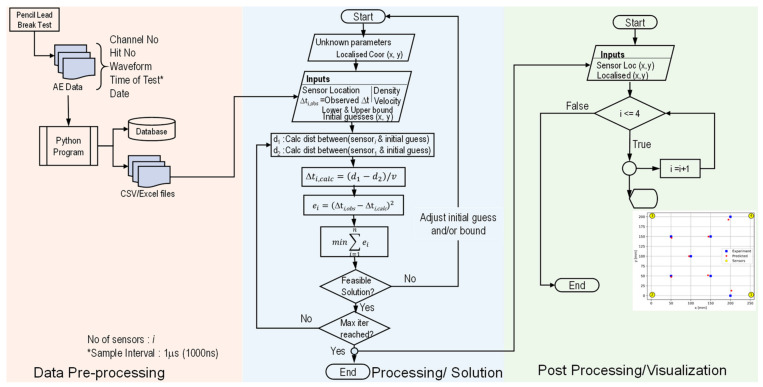
A detailed flow chart depicting TDOA localization technique.

For the ideal case, χ^2^ = 0 at the source location. However, for real cases, the sum is recalculated for each potential source location x_s_ and y_s_ that minimize the value of χ^2^. The lower the value, the better a model fits a dataset. A detailed flowchart of the above process, along with an example AE source localization, is shown in Figure 3.

## 4. Acoustic Emission Frequencies and Velocity Profiles for Anisotropic Materials

For non-homogeneous composite structures, the assumptions made in the earlier section are far from true because of anisotropic material properties, which lead to varying acoustic wave velocity. In fact, the acoustic wave velocity for the composite laminates can be a function of wave frequency, laminate thickness, manufacturing quality, as well as the mechanical properties of the laminate itself. In addition, although the assumption that AE waves travel in a straight line from the AE source to the sensors is mostly valid for uniform thickness panels, it would be untrue in the case where the geometry has voids, discontinuities, or cutouts. 

Because of the above reasons, the waveforms detected by each AE sensor might be quite different from each other. Acoustic waves have a wide frequency spectrum, ranging from <1 Hz in earthquakes to several MHz in metals and ceramics. For typical AE testing, a frequency above 20 kHz is used to exclude environmental and machinery noise and the human voice. Typical frequency ranges for composites and metals and ceramics are 20–100 kHz and 100–500 kHz, respectively [5].

**Figure 4:**
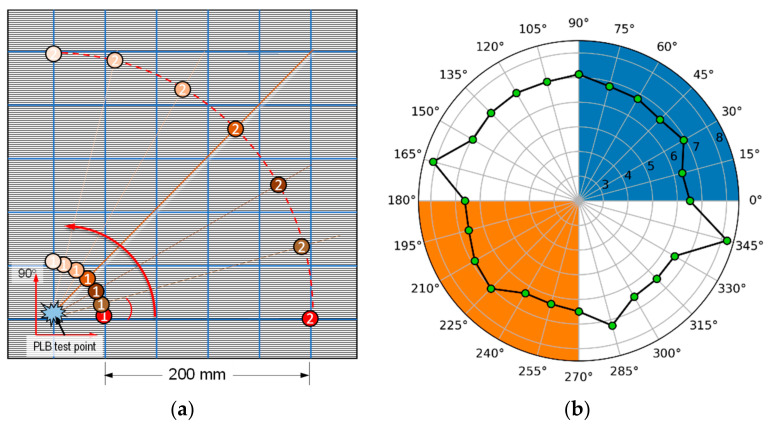
(**a**) Experiment layout for determining the AE velocity for 200 mm distance and (**b**) velocity profile measured at 200 mm between the sensors.

Acoustic properties primarily vary with the material and geometry of the structure, and, thus, testing is generally recommended on the actual specimen to determine acoustic velocities and attenuations [5]. In order to make an accurate measurement of AE velocities, PLB tests were conducted on the composite panel for the measurements of the AE wave velocity at a distance of 200 mm from the first sensor, with a 360° rotation and a 15° step, as shown in Figure 4a. The results of three tests were averaged to calculate the velocity, and a corresponding plot was generated, as depicted in Figure 4b. The number 3, 4, 5, 6, 7, and 8 in Figure 4b represents the corresponding velocity in ×1000 m/s.

An intriguing observation was made in the velocity profile, wherein the highest velocity was found to be at −15° (or 345°) and 165°, angles that are 180° apart. It is widely acknowledged that a higher acoustic wave propagation occurs in the fiber orientation in comparison to other angles. One possible reason for this phenomenon could be attributed to the slight clockwise orientation of the top lamina.

The AE wave velocity at the proximity of the PLB test is high, and as the distance from the AE event source increases the velocity decays. The pattern of this decay was observed to be exponential decay over time and distance. In order to accurately determine the attenuation pattern, additional tests were performed. Three test points were selected at 100 mm, 150 mm, and 200 mm from the origin, and also, for each test point, three PLB tests were repeated. The average wave velocities at 100 mm, 150 mm, and 200 mm were found to be 10,205.6 m/s, 7860.6 m/s, and 7448.7 m/s, respectively. While velocity attenuation appears to occur at a faster rate, previous studies [40,48,49] examining stress wave velocity attenuation have yielded comparable results. The observation aligns with the findings of Benmedakhene and Laksimi [41], where they showed wave propagation velocity exhibited an exponential decay.

**Figure 5:**
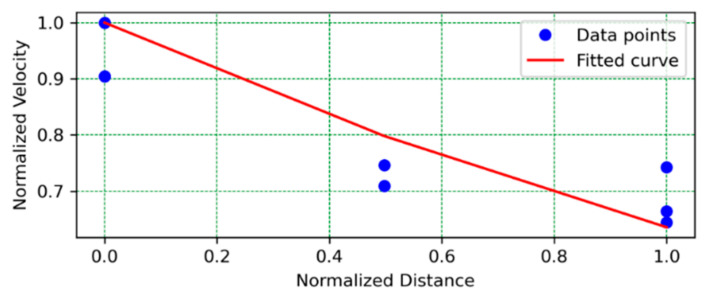
Exponential decay function as a function of x–y distance and AE signal velocity for 15°.

Since the magnitude of the velocity (1 × 10^4^ m/s) and distance (1 × 10^−3^ m) are several order magnitude differences, the values are normalized to ensure meaningful comparison and analysis. A representative exponential AE wave decay is shown in Figure 5. The knowledge of attenuation patterns can also help suggest the number of sensors and sensor separation distance for a test specimen.

The curve_fit() function from the SciPy optimize module was used to fit the curve and calculate the exponential time constant for all the experimental values that included velocity (m/s) and distance (mm) for experiments performed between 0 and 90° angles with 15° step. Table 1 lists the exponential decay constant for the AE wave profile with corresponding angles.

**Figure 6:**
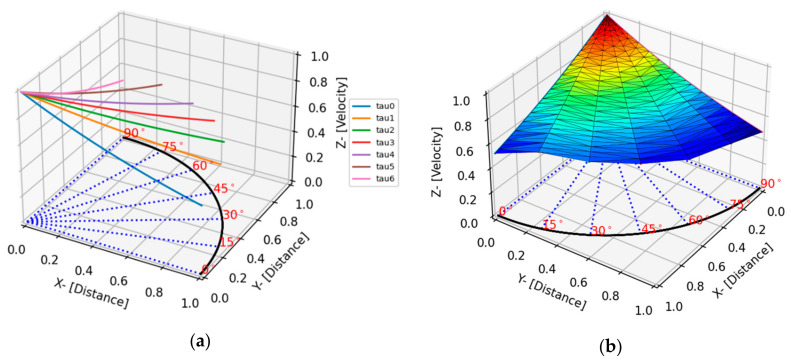
(**a**) Velocity attenuation profile at various angles and (**b**) the surface plot for visualizing the response surface of the AE velocity attenuation.

The obtained decay constant was further used in the exponential decay function (Equation (7)) to generate an exponential profile.

(7) $$\hat{v}=e^{\left(-\frac{\hat{d}}{\tau }\right)}$$

where $\hat{d}$ is a normalized distance, *τ* is a decay constant and $\hat{v}$ is normalized velocity. Based on the experimental data and the exponential decay function a 3D plot was generated, as shown in Figure 6a. To include this information in a localization program, a separate function was developed to visualize the response surface that takes velocity attenuation into consideration. A tri-surface plot using plot_trisurf() function in Matplotlib was used to generate a surface plot. Under the hood, plot_trisurf() function uses the Delaunay triangulation algorithm to create a set of triangular facets from the input points, following which it creates a surface. Figure 6b on the right is rotated along the *z*-axis for better visualization.

## 5. Localization Algorithm

**Figure 7:**
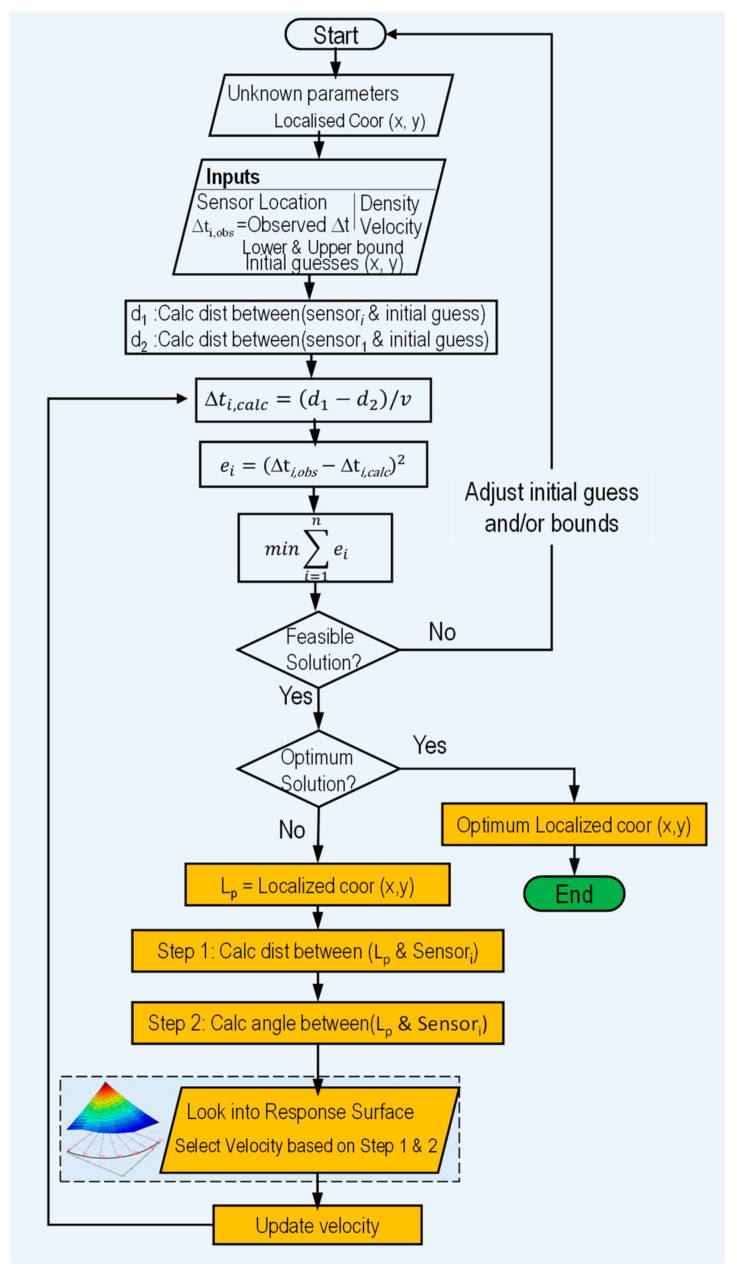
A flowchart of the COLORS algorithm showing the response surface and updated velocity for locating the AE source.

The COLORS (COmposite LOcalization using Response Surface) algorithm has been developed and implemented in Python in order to accurately locate the AE source in the composite laminates. The in-house program written in Python expects maximum AE wave velocity instead of theoretical AE velocity, as typically used in the TDOA method. The program automatically calculates velocity at a specific distance and angle based on the response surface discussed in the previous section. The maximum velocity can be determined in two ways; (a) experimentally obtained through PLB tests and (b) it can be calculated by extrapolating the AE wave velocity using a known decay constant and intermediate AE velocity data. The flow chart of the proposed algorithm is shown in Figure 7.

**Figure 8:**
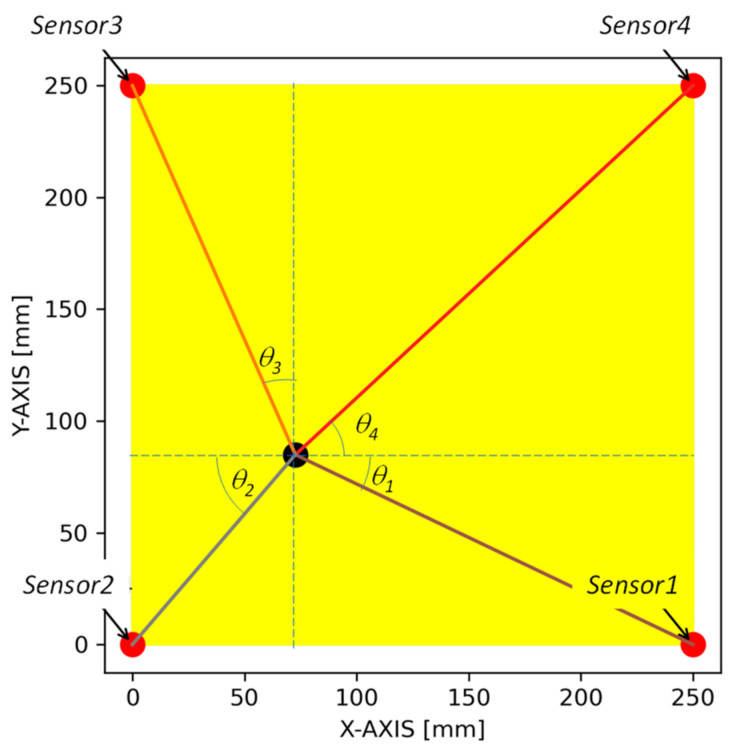
Angles and distances calculated from the approximate the AE event location determined by the TDOF algorithm.

In order to quantitatively determine the exact velocity, the program runs to determine the approximate the AE event location using the TDOA algorithm described in the aforementioned section. Based on the approximate AE location, the angles (θ_1_, θ_2_, θ_3_, and θ_4_) and distance (d_1_, d_2_, d_3_, and d_4_) were calculated for each sensor, as shown in Figure 8. Once all the angles to the sensors and corresponding distances were determined, they were further mapped to the response surface to accurately determine the AE wave velocity. The novel algorithm uses the updated velocity to determine the AE event location.

After determining the velocity for each sensor by considering its orientation and approximate distance through the response surface, the program iterates by rerunning with the updated wave velocity. Subsequently, the residual is calculated by employing the minimize() function of SciPy utilizing a modified Powell algorithm to minimize the objective function. The entire process is systematically replicated for subsequent AE events.

## 6. Results and Discussion

**Figure 9:**
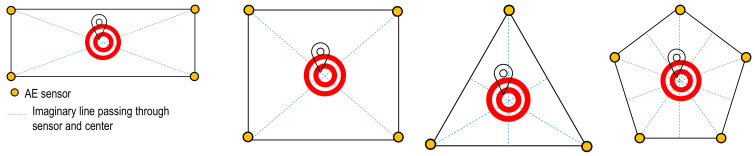
Example of special cases where the TDOA method can be reasonably used for localization in composite structures.

The TDOA algorithm is a versatile algorithm; and primarily finds application in localization problems within homogenous and isotropic materials. The TDOA method relies on a constant AE wave velocity for the triangulation technique. Despite this, TDOA algorithm also proves valuable for addressing specialized localization problems in laminated composite structures. One such distinctive scenario involves a 1D localization problem, where sensors align linearly, exhibiting a symmetrical distribution on both sides of an AE event. Other special cases include situations where the AE events occur precisely at or in close proximity to the central point of the sensor network, as illustrated in Figure 9.

**Figure 10:**
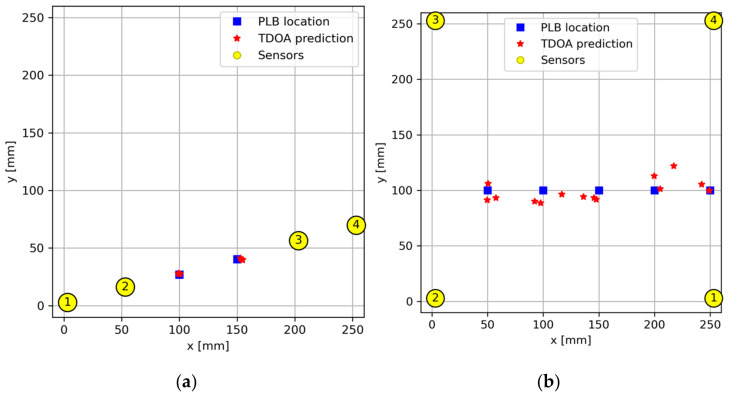
AE source location using TDOA method (6150 m/s) for special cases (**a**) a linear (or 1D) sensor arrangement and (**b**) near-center AE events.

Figure 10 displays the outcomes of both scenarios conducted on CFRP composite panels. It can be seen that the TDOA method, using the proper minimization algorithm, yields predictions closely aligned with the actual occurrence of AE events.

However, the TDOA method encounters challenges in accurately locating AE events within anisotropic materials and the predicted location exacerbates as the deviation from special cases increases. Implementing the TDOA method in composite materials presents an additional challenge related to the selection of the AE wave velocity. The inherent anisotropic properties of composite materials make it challenging to determine an accurate AE wave velocity, as it depends on factors such as elastic modulus, which further relies on fiber direction, density, and thickness [49].

**Figure 11:**
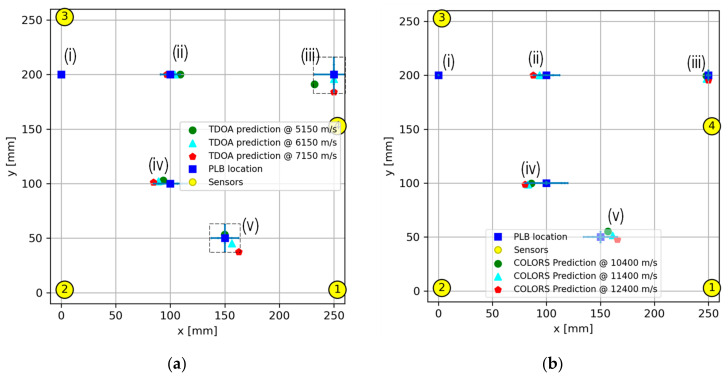
AE localization result using (**a**) average AE wave velocity and (**b**) novel COLORS algorithm using updated velocity from the response surface.

To address these challenges, a comparative study was conducted to determine the most suitable AE wave velocity among three velocities (5150 m/s, 6150 m/s, and 7150 m/s), representing the typical range for common carbon fiber-reinforced epoxy composites. Figure 11a illustrates the results of this study, with specific values chosen in 1000 m/s increments for ease of comparison. Through repeated experiments, it was revealed that an AE wave velocity of 6150 m/s gives reasonable accuracy for the composite panel used in the study.

In contrast, the COLORS algorithm, as an alternative to using a constant AE wave velocity, incorporates an accurately determined AE wave velocity by mapping the approximate orientation and distance predicted from the TDOA method. The localization results using the proposed COLORS algorithm are depicted in Figure 11b, showcasing resilience and nearly accurate predictions even when the maximum velocity deviates by 1000 m/s. This resilience is attributed to the algorithm’s adaptive nature, utilizing only a fraction of the maximum velocity in the optimization process, dependent on the specific point on the response surface.

Having precisely determined the AE source through a comparison of three velocities, it was found that employing a velocity of 11,400 m/s yielded the most favorable result. Notably, minor discrepancies in the maximum velocity were found to have minimal impact on the accuracy of predicting the AE event locations.

**Figure 12:**
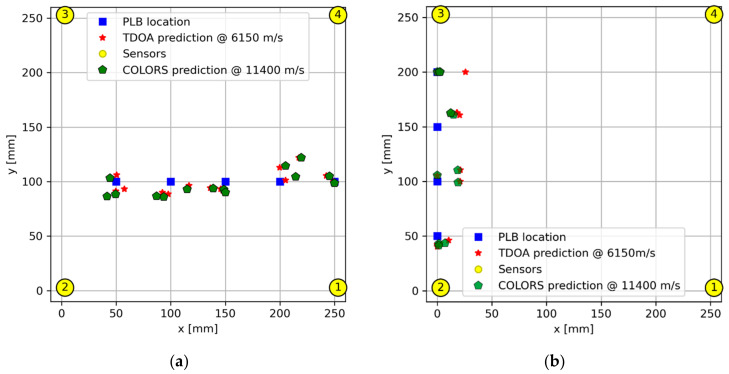
Case studies for comparing the TDOA method and COLORS algorithm for (**a**) near center AE event for the sensor network and (**b**) extreme edge of the sensor network.

To validate the efficacy of the COLORS algorithm, two distinct cases were subjected to validation tests. The first case involved an AE event occurring in close proximity to the center, where the COLORS algorithm demonstrated comparable accuracy in predicting the AE event location as the TDOA method, as depicted in Figure 12a. In the second case, with the AE event taking place at the extreme corner of the sensor network, the COLORS algorithm outperformed the TDOA method significantly. The TDOA method exhibited a deviation towards the right as the AE event moved further up, while the COLORS algorithm maintained superior prediction accuracy, as visually confirmed in Figure 12b.

Furthermore, to quantitatively assess the performance, mean absolute error (MAE) and root mean squared error (RMSE) were computed using Equations (8) and (9).

(8) $$MAE=\frac{1}{n}\overset{n}{\underset{i=1}{\sum }}\left|x_{i}-y_{i}\right|$$

where *n* is the number of predictions, *x_i_* is the prediction of the algorithm (TDOA or COLORS) and *y_i_* is the corresponding true value.

(9) $$RMSE=\sqrt{\frac{1}{2}\overset{n}{\underset{i=1}{\sum }}{\left(x_{i}-y_{i}\right)}^{2}}$$

The MAE values for COLORS and TDOA were determined to be 6.97 mm and 8.69 mm, respectively. Similarly, the RMSE values for COLORS and TDOA method were found to be 9.24 and 12.06, respectively, indicating the superior performance of the COLORS algorithm in terms of the AE source location accuracy. The detailed performance metrics are summarized in Table 2. This validation underscores the COLORS algorithm’s enhanced capability for general AE localization compared to the TDOA method.

## 7. Conclusions

In this study, PLB tests were carried out to comprehensively characterize the acoustic wave velocity propagation and attenuation in CFRP laminated panels. The exploration of intricate patterns of the AE wave velocity in anisotropic materials, with a focus on higher velocities along the fiber orientation, led to the investigation of the relationship between AE wave velocity, laminate orientation, and distance. The experimental results were leveraged to generate a response surface, serving as a mapping tool for accurately determining acoustic wave velocity based on orientation and distance. Building upon this foundation, the COLORS algorithm was developed to incorporate variable AE wave velocities.

A rigorous comparative analysis with the widely used TDOA method demonstrated the clear superiority of the COLORS algorithm in precisely pinpointing AE event locations within composite structures. This represents a significant advancement in the field, offering a promising avenue for enhancing damage localization in such structures. Damage localization in this context refers to the process of identifying the precise location of damage within the composite panel, typically expressed in terms of spatial coordinates (e.g., millimeters). The main conclusions include:

1. The TDOA method’s assumptions of constant AE wave speed and a straight-line path between the sensor and AE source are invalidated for anisotropic materials like CFRP laminates. Experimental results allowed us to determine the acoustic wave velocity profile and attenuation rate for laminate orientation, enabling the creation of a response surface. The response surface acts as a mapping tool, accurately determining acoustic wave velocity based on orientation and distance, thus reducing testing time and cost.
2. The COLORS algorithm employs a two-step process for accurately predicting the AE source localization. Initially, it utilizes a constant velocity and the TDOA method to approximate the AE location. Subsequently, it calculates the orientation and distance to each sensor, refining the acoustic wave velocity based on the response surface.
3. The acoustic velocity from the response surface is updated and integrated into the COLORS algorithm, significantly improving acoustic localization in composite structures.
4. While testing and validation were conducted on a relatively small composite panel, the proposed technique holds the potential for localizing damages in larger composite structures. The response surface accounts for the acoustic wave attenuation rate, which is particularly dominant in large structures with sparsely located AE sensors.

In summary, this study demonstrates the accurate determination of AE wave velocity in composite structures using a response surface. The novel COLORS algorithm, introduced here, capitalizes on this response surface to extract velocity information for each AE signal based on orientation and distance from the approximate localized point using the TDOA method. However, to fully understand the algorithm’s potential, comprehensive experiments encompassing diverse material properties, panel sizes, and conditions are necessary. The authors believe that the findings of this research will significantly impact AE-based research, finding wide applications in both industry and academia.

## 8. Limitations and Future Study

Although the results from the study are promising and show significant improvement in the AE localization problem in composite laminates, there are some limitations to the present studies.

Firstly, the laminated composite panel used in this study was a simple, uniform, thickness panel. Further experiments are planned to benchmark the validity of the developed code on non-uniform panels with varied lamination sequences.

Secondly, the current study noted significant deviations in some coordinate values, particularly the x-coordinates, when using the TDOA and COLORS methods. These deviations can be attributed to factors such as sensor placement accuracy and signal propagation characteristics. While MAE and RMSE metrics provide a comparative basis for evaluating the methods, future studies will delve deeper into the reasons behind these deviations, especially in larger panels and more complex scenarios.

Thirdly, real structures can have complex geometries with cutouts and stiffeners, which modify the AE waveform characteristics. Thus, applying the proposed techniques to more complex composite structures will be the subject of future studies.

Finally, by deploying the proposed techniques in a real structure, more insight can be received, providing further insights into how to proceed with improvements. Future studies will focus on this practical deployment to gather more data and refine the techniques accordingly.

## Figures and Tables

**Table 1:** Exponential decay time constant obtained from the experiment for 0° to 90° (at 15° intervals).

Angle (°)	Decay Constant (τ)
0	1.533
15	2.206
30	1.997
45	1.822
60	1.635
75	1.668
90	1.352

**Table 2:** True and predicted coordinate using TDOA and COLORS algorithm and corresponding MAE and RMSE values.

TRUE (Coordinate)		TDOA (Coordinate)		COLORS (Coordinate)	
X	Y	X	Y	X	Y
0	50	0	42.16	1.52	42.87
0	50	10.67	46.14	7.03	43.55
0	50	0	40.29	1.4	41.21
0	100	20.99	100.01	19.15	99.06
0	100	21.24	110.37	18.8	110.12
0	100	0	105.24	0	105.62
0	150	17.89	162.75	12.22	162.39
0	150	20.57	160.59	14.97	160.78
0	150	18.24	162.75	12.59	162.53
0	200	0.03	199.99	0	199.94
0	200	25.82	199.99	2.82	199.99
0	200	0.06	199.99	2.09	199.99
MAE		8.69 mm		6.97 mm	
RMSE		12.06 mm		9.24 mm	

## Data Availability

The raw data required to reproduce these findings are available from the authors upon formal request. However, the computer program developed in this study is currently unavailable as it has been lodged for invention disclosure. Due to confidentiality and intellectual property considerations, the program cannot be shared or distributed at this time.
